# Optimal Stenting Strategy During Chemotherapy: Impact of Time to First Reintervention on Survival in Malignant Hilar Biliary Obstruction

**DOI:** 10.1002/deo2.70351

**Published:** 2026-05-23

**Authors:** Katsuhiko Sato, Minoru Shigekawa, Teppei Yoshioka, Kazuma Daiku, Syuhei Yamamoto, Yu Sato, Takahiro Kodama, Hayato Hikita, Tetsuo Takehara

**Affiliations:** ^1^ Department of Gastroenterology and Hepatology The University of Osaka Graduate School of Medicine Osaka Japan; ^2^ Department of Hepatobiliary and Pancreatic Oncology Osaka International Cancer Institute Osaka Japan

**Keywords:** biliary tract neoplasms, cholangiocarcinoma, cholestasis, drug therapy, stents

## Abstract

**Objectives:**

Advances in chemotherapy have increased survival in biliary tract cancer patients, and reports of the effectiveness of neoadjuvant therapy are emerging. Accordingly, developing optimal drainage strategies under chemotherapy is important. We aimed to clarify suitable drainage methods and assess the prognostic value of the time to first reintervention (TTFR) following chemotherapy induction.

**Methods:**

We retrospectively analyzed 81 patients with malignant hilar biliary obstruction (MHBO) who underwent biliary stenting followed by chemotherapy between April 2012 and October 2023. TTFR following chemotherapy induction and drainage‐ and survival‐related factors were evaluated.

**Results:**

The median follow‐up, survival, and TTFR were 425, 479, and 167 days, respectively. TTFR was correlated with overall survival (*r* = 0.60, *p* < 0.01). Univariable analyses identified prechemotherapy cholangitis and stenting above the sphincter of Oddi (SO) as factors associated with shorter TTFR, whereas multivariable analysis revealed stenting above the SO as the only independent factor (hazard ratio [HR] 0.44, *p* = 0.01). Among 52 non‐endoscopic sphincterotomy (EST) patients, prechemotherapy cholangitis (HR 2.05, *p* = 0.04), stenting above the SO (HR 0.47, *p* = 0.04), and multiple drainage segments (HR 0.42, *p* = 0.04) influenced TTFR in univariable analyses. No significant factor was detected among 28 patients undergoing EST. The TTFR was comparable between above‐ and across‐SO placement groups up to 2 months, but superior in the above‐SO group at 6 months (69%–73% vs. 27%–42%).

**Conclusions:**

TTFR following chemotherapy induction is a prognostic factor in MHBO patients. Stenting above the SO may prolong TTFR, particularly in those without prior EST or expected to continue chemotherapy beyond 2 months.

**Trial Registration: N/A**.

## 1 Introduction

Malignant hilar biliary obstruction (MHBO) is often associated with jaundice at the time of diagnosis and requires biliary drainage. However, the optimal endoscopic drainage strategy for patients with MHBO remains unclear. Indications for surgery differ from institution to institution. Unilateral drainage of the residual lobe, preferably using plastic stents, is recommended for patients intended for surgery [1, 2]. For patients undergoing palliative therapy, it is considered desirable to secure larger drainage areas with bilateral drainage to help preserve liver function [3]. Metal stents are reported to be superior to conventional plastic stents when placed across the sphincter of Oddi (SO) [4, 5]. Another report suggested that when placed above the SO, plastic stents and metal stents are of equal value [6]. In clinical practice, stenting strategies for MHBO vary across institutions.

Resection of perihilar cholangiocarcinoma, a representative form of MHBO, is associated with high mortality, resulting in a 5‐year survival rate of less than 50% [7, 8]. In a study comparing neoadjuvant and adjuvant chemotherapy in biliary tract cancers, neoadjuvant chemotherapy (NAC) was preferred in intrahepatic locations, and patients who received NAC had longer overall survival (OS) (40.3 vs. 32.8 months, *p* = 0.01) and a higher percentage of R0 resection than those who received surgery upfront did (71% vs. 61%, *p* = 0.02) [9]. In intrahepatic cholangiocarcinoma, the treatment of locally advanced unresectable cancers with NAC, mainly gemcitabine and oxaliplatin, yielded a disease control rate of 70% after surgical resection, which was similar to that of resectable cancers [10]. Immune checkpoint inhibitors (ICIs), namely, pembrolizumab [11] and durvalumab [12], have extended OS compared with the combination of gemcitabine and cisplatin, which was the standard chemotherapy for more than a decade [13]. Although data on NAC remain limited, ICIs may expand surgical eligibility in patients with MHBO. This would result in an increased number of patients undergoing chemotherapy with biliary stent placement, preferably using plastic stents, with the intention of surgical resection.

The use of plastic stents placed above the SO may be a good option for patients with MHBO in whom surgical resection is possible. The deployment of plastic stents above the SO has been shown to improve the stent patency time compared with their placement across the SO [4]. Endoscopic sphincterotomy (EST) is widely performed for easier cannulation of the biliary duct and to reduce the risk of post‐endoscopic retrograde cholangiopancreatography (post‐ERCP) pancreatitis [14]. However, few studies have focused on EST and its relationship with stenting above the SO in the context of targeting MHBO. Considering that the advantage of stenting above the SO lies in preventing the reflux of food components into the stent, post‐EST patients, especially those who are undergoing chemotherapy and prone to cholangitis, may not benefit from this approach.

In this study, we focused on the time to first reintervention (TTFR) from chemotherapy induction. We analyzed the relationship between the TTFR and the stenting method in patients with MHBO undergoing chemotherapy, with or without EST, to identify the optimal stenting approach for patients with MHBO undergoing chemotherapy in the upcoming era of NAC.

## 2 Methods

### 2.1 Patients

**FIGURE 1:**
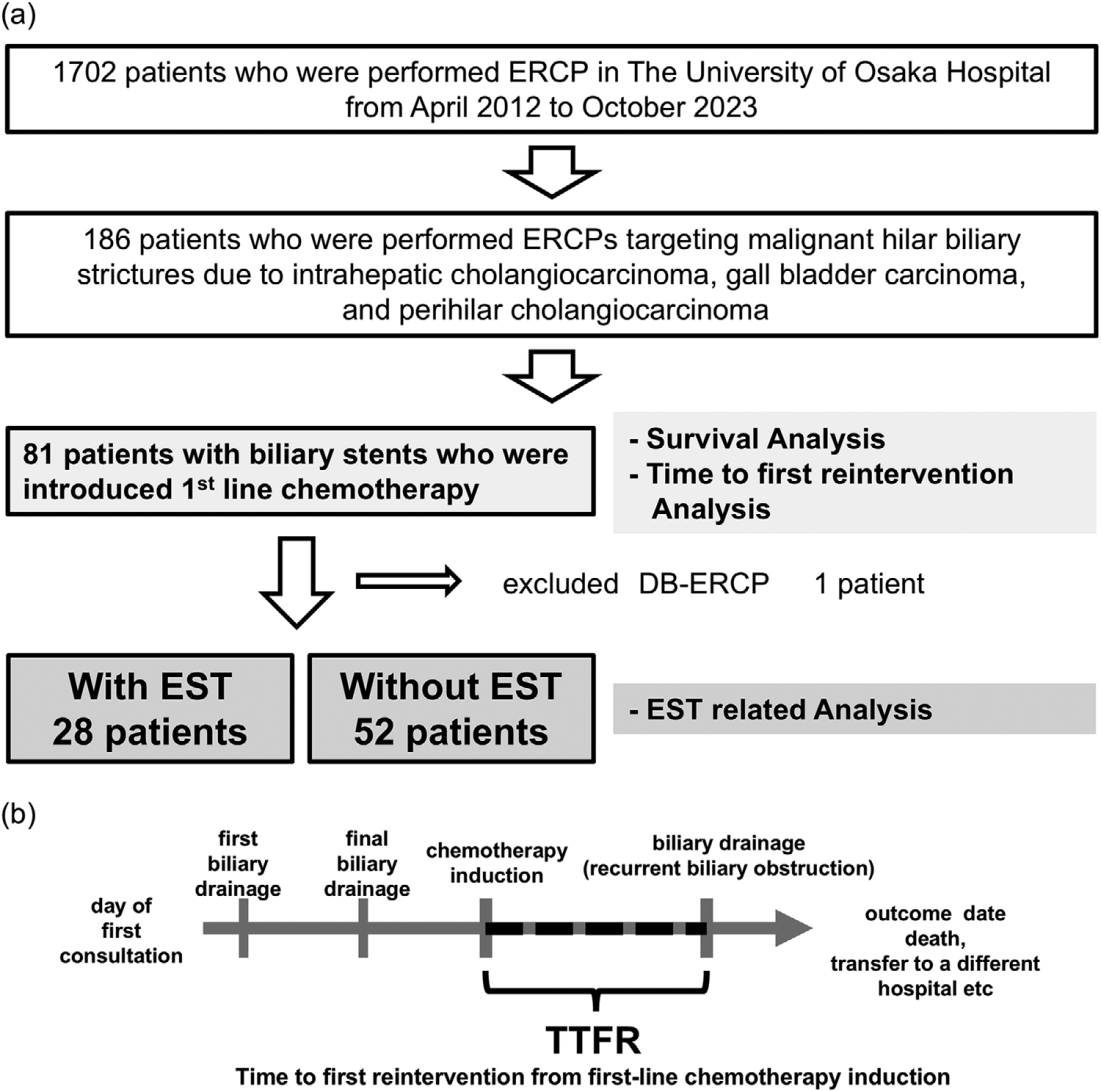
Patients enrolled in the study and definition of the time to first reintervention (TTFR) from first‐line chemotherapy induction. (a) Patients enrolled in the study. (b) Definition of the time to first reintervention (TTFR) from first‐line chemotherapy induction.

Among the 1702 consecutive patients who underwent ERCP at The University of Osaka Hospital from April 2012 to October 2023, 186 consecutive patients who underwent ERCP targeting MHBO due to intrahepatic cholangiocarcinoma, gallbladder carcinoma, and perihilar cholangiocarcinoma were identified from medical conference records. From this cohort, 81 patients with biliary stents who were treated with first‐line chemotherapy were identified for analysis. The TTFR and OS were analyzed in this group. For EST‐related analyses, one patient who underwent double balloon‐ERCP with no papilla was excluded. Twenty‐eight patients treated with EST and 52 patients not treated with EST were included in the EST‐related analysis (Figure 1a). All patients underwent an expert multidisciplinary cancer board conference before and after ERCP to discuss the diagnosis and treatment options, including stenting. Our strategy for identifying suspected MHBO is described below. Dynamic computed tomography (CT) and/or magnetic resonance imaging (MRI) were performed prior to the expert conference. ERCP was performed with the aim of achieving biliary drainage and a pathological diagnosis (Figure S1). When patients were considered candidates for surgical resection, either upfront or after chemotherapy, a mapping biopsy was performed to assess the extent of bile duct invasion. We divided the liver into three segments, namely, the left lobe, anterior segment, and posterior segment, and addressed each segment as being of equal value within this study. Drainage of the planned residual lobe with a nasobiliary tube was performed, and after confirming the preservation of liver function and control of cholangitis, a plastic stent deployed above the SO was placed in the previously drained area. In unresectable patients, the drainage method was decided in an expert conference. Drainage of one segment was selected if more than 50% of the liver volume could be saved with one stent. In cases where jaundice did not improve with drainage of one segment or drainage of half the liver volume could not be achieved with drainage of one segment, drainage of multiple segments was selected (Figure S1). EST was not routinely performed for patients with MHBO; however, some patients had undergone EST at another hospital before referral during the initial drainage procedure or prior to the suspicion of biliary tract cancer.

### 2.2 ERCP‐related Methods

ERCP was performed using a JF‐260V, TJF‐260V, or TJF‐290V scope (Olympus Medical Systems, Tokyo, Japan). Plastic stents deployed above the SO included the Advanix J (Boston Scientific, Marlborough, MA, USA) and Through & Pass (Gadelius Medical, Tokyo, Japan) stents. Stents deployed across the SO included the Flexima (Boston Scientific), Through & Pass (Gadelius Medical), and Zimmon (Cook Medical, Bloomington, IN, USA) stents.

### 2.3 Definitions

Clinical success was defined as a reduction in total bilirubin by half or a normal bilirubin level within 14 days of drainage according to the Tokyo criteria 2024 [15]. Post‐ERCP pancreatitis was defined as an amylase level of three times the upper limit or more with concomitant abdominal pain and cholangitis [16]. Cholangitis was assessed according to Tokyo guidelines 2018 as follows: inflammation (physical examination/laboratory data), cholestasis (laboratory data), and imaging (biliary duct dilatation, stent deployment, etc.) [17]. OS was defined as the time from the first consultation day to the outcome date. The TTFR was defined as the time from the first day of chemotherapy to the first reintervention ERCP (Figure 1b).

### 2.4 Outcome Measurement

The primary goal was to identify stenting‐related factors influencing the TTFR in patients with MHBO with biliary stents following the initiation of first‐line chemotherapy. The secondary goals were to assess the effect of the TTFR on OS and to analyze whether EST affects the selection of the optimal stenting method in patients with MHBO undergoing chemotherapy.

### 2.5 Statistical Analysis

The data are presented as medians with ranges. The chi‐square test was used for categorical variables, while the Mann–Whitney U test was used for continuous variables. The median values were used as cutoff points for continuous variables, including neutrocyte‐to‐lymphocyte ratio and TTFR. A Cox regression model was used to analyze factors associated with OS and TTFR. The Kaplan–Meier method was used for survival curve analysis. Statistical analyses were performed using JMP Pro 17 software (SAS Institute, Cary, NC, USA).

## 3 Results

### 3.1 Patient Characteristics at Diagnosis and Prior to the Initiation of First‐line Chemotherapy

Among the 81 patients with biliary stents who underwent chemotherapy (four patients with intrahepatic cholangiocarcinoma, 17 patients with gall bladder carcinoma, and 60 patients with perihilar cholangiocarcinoma), 54 were male, and the median age was 72.6 years. Clinical metastasis was detected in 35 patients at chemotherapy induction. The median tumor marker levels were 3.0 ng/mL for CEA and 250 U/mL for CA19‐9. In all, 22 patients received NAC, 59 received palliative chemotherapy, and 15 underwent surgery after chemotherapy (Table 1). Cholangitis before chemotherapy induction was detected in 48 patients, and 28 patients underwent EST. There were 47 patients with drainage of one segment at chemotherapy induction. Stenting above the SO was performed in 33 patients, whereas stenting across the SO was performed in 46 patients. The median follow‐up period was 425 days. Unscheduled reintervention was required in 56 patients. Reasons included 6 tumor‐related and 44 non‐tumor‐related factors; data were insufficient for analysis in six patients (Table 1). Post‐ERCP pancreatitis occurred in 9 patients but did not delay chemotherapy induction (Table S3).

**TABLE 1:** Patient baseline characteristics.

Variable		All Patients (*n* = 81)	Variable		All Patients (*n* = 81)
Sex, *n*	male/female	54/27	First‐line chemotherapy, *n*	GCD/GCS/GC/GS/GEM/S1/other	4/20/36/1/10/4/6
Age, years	median (range)	72.6 (38–87)	Type of chemotherapy, *n*	neoadjuvant/palliative	22/59
ASA PS, class	1/2/3	37/37/7	Surgery after chemotherapy, *n*	yes/no	15/66
Bismuth‐Corlette type, *n*	I/II/IIIa/IIIb/IV	17/12/29/7/16	Clinical success^b^, *n*	yes, % (*n*)	79% (61/77)
Clinical metastasis^b^, *n*	M0/M1	45/35	Cholangitis before chemotherapy induction, *n*	yes/no	48/33
ALT^b^, IU/L	median (range)	32 (5–136)	EST (including previous), n	yes/no/choledochojejnunostomy	28/52/1
GGT^b^, IU/L	median (range)	226 (15–1154)	ENBD before chemotherapy induction, n	yes/no	54/27
T‐Bil^b^, mg/dL	median (range)	1.0 (0.3–3.5)	Drainage method at chemotherapy induction, *n* ^a^	ENBD/PS/inside stent/SEMS/PTBD	1/43/22/11/4
Albumin^b^, g/dL	median (range)	3.7 (2.2–4.6)	Drainage segments at chemotherapy induction, *n*	1/2/3	47/27/7
Neutrophil‐to‐lymphocyte ratio^b^, A.U.	median (range)	2.8 (0.2–26.1)	Stenting above vs across the Sphincter of Oddi, *n*	above/across/other	33/46/2
CEA^b^, ng/mL	median (range)	3.0 (1–1024)	Post‐ERCP pancreatitis, *n*	no/mild/moderate/severe/no data	70/3/5/1/2
CA19‐9^b^, U/ml	median (range)	250.0 (0.4–407606.4)	Unscheduled reintervention, *n*	yes/no	56/25
Follow‐up period, days	median (range)	425 (54–2859)	Reason for re‐intervention, *n*	over or ingrowth/food debris/sludge/stent dislocation/no data	6/12/29/3/6

ALT, alanine transaminase; ASA‐PS, American Society of Anesthesiologists physical status; CA19‐9, carbohydrate antigen 19‐9; CEA, carcinoembryonic antigen; GC, GEM+CDDP; GCD, GEM+CDDP+Durvalumab; GCS, GEM+CDDP+S1; GGT, gamma‐glutamyltransferase; GS, GEM+S1; ENBD, endoscopic nasobiliary drainage; ERCP, endoscopic retrograde cholangiopancreatography; EST, endoscopic sphincterotomy; PTBD, percutaneous transhepatic biliary drainage; PS, plastic stent deployed transpapillary; SEMS, self‐expandable metallic stent; T‐Bil, total bilirubin.

^a^ ENBD+X→ENBD, PTBD+X→PTBD, PS+inside→PS.

b Patients without data are excluded.

### 3.2 The TTFR From First‐line Chemotherapy Induction Was a Prognostic Factor Correlated With OS

**FIGURE 2:**
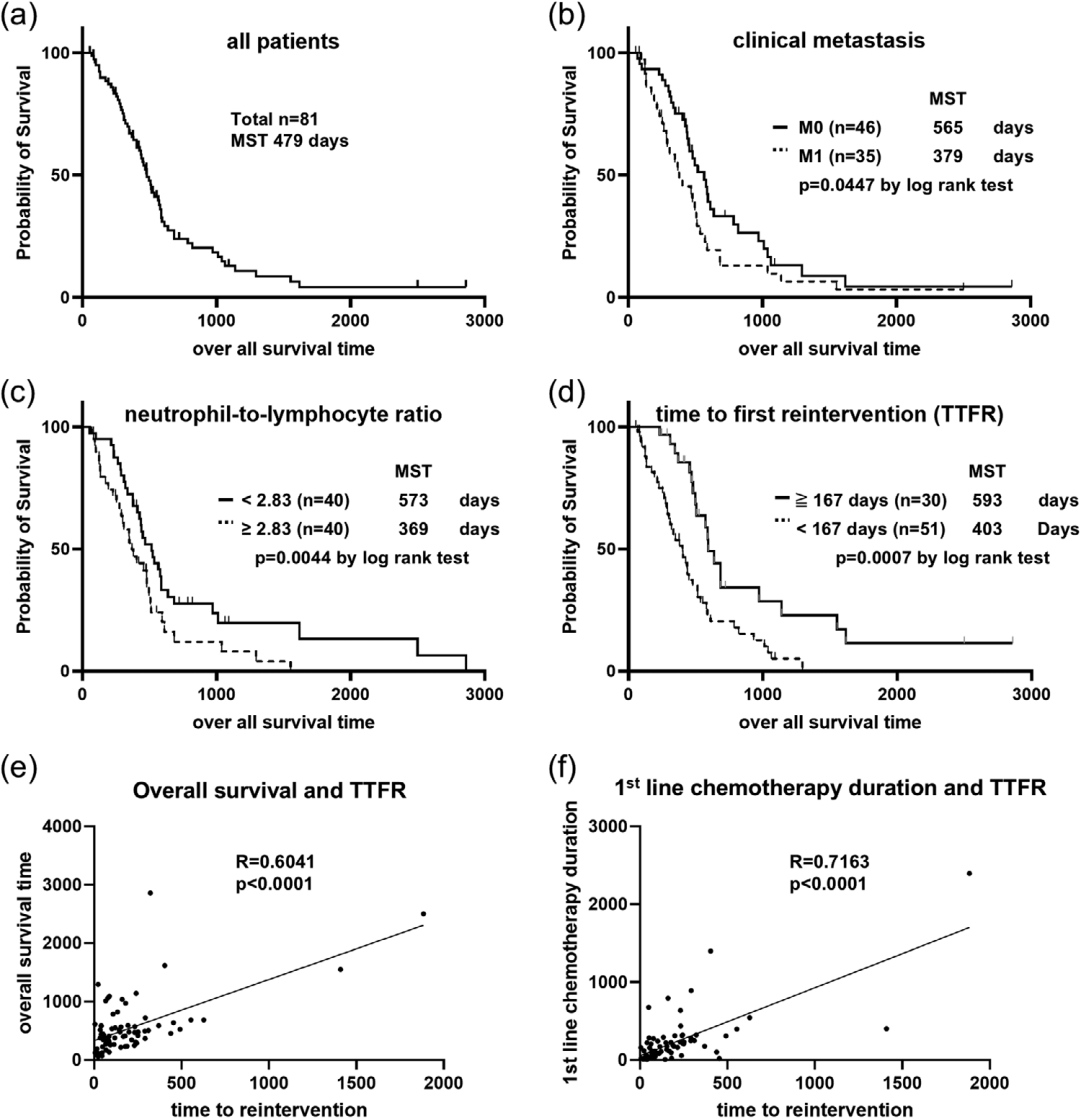
Factors related to overall survival. Overall survival (OS) of 81 patients with malignant hilar biliary obstruction (MHBO) according to the Kaplan–Meier method. (a) The median survival time (MST) was 479 days. (b) Patients with clinical metastasis had poorer OS than those without metastasis (MST: 379 days vs. 565 days, *p* = 0.0447, log‐rank test). (c) Patients with a high neutrophil‐to‐lymphocyte ratio (NLR) had a shorter OS than those with a low NLR (MST: 369 days vs. 573 days, *p* = 0.0044, log‐rank test). (d) Patients with a longer time to first reintervention (TTFR) from first‐line chemotherapy induction had a longer OS than those with a shorter TTFR (MST: 593 days vs. 403 days, *p* = 0.0007, log‐rank test). (e) The TTFR was correlated with OS according to Pearson's correlation (*r* = 0.6041, *p* < 0.0001). (f) The TTFR was correlated with the duration of first‐line chemotherapy according to Pearson's correlation (*r* = 0.7163, *p* < 0.0001).

The median survival time (MST) of all 81 patients was 479 days (Figure 2a). Univariate analysis revealed that a high neutrophil‐to‐lymphocyte ratio (NLR), clinical metastasis, and short TTFR were factors related to a poor prognosis (Table 2). Multivariate analysis of these three factors and Bismuth–Corlette types IIIa+IV revealed the NLR (hazard ratio [HR] 2.13, *p* = 0.006), clinical metastasis (HR 2.32, *p* = 0.004), and the TTFR (HR 0.27, *p* = 0.001) as factors related to OS (Table 2). No endoscopy‐related factors, including EST or stenting method (above or across the SO), affected prognosis (Table 2). A lower NLR (573 days vs. 369 days, *p* = 0.0044), absence of clinical metastasis (565 vs. 379 days, *p* = 0.045), and a longer TTFR (593 vs. 403 days, *p* < 0.001) prolonged the MST (Figure 2b–d). The TTFR was correlated with OS (*r* = 0.6041, *p* < 0.001) and the first‐line treatment duration (*R* = 0.7163, *p* < 0.001) (Figure 2e,f).

**TABLE 2:** Risk factors associated with overall survival.

Variable			Univariate analysis			Multivariate analysis		
		*n*	HR	95% CI	*p*‐Value	HR	95% CI	*p*‐Value
Sex	male	54	1.08	0.630–1.860	0.773			
	female	27	1					
Age	≥75 years	32	0.79	0.473–1.313	0.361			
	<75 years	49	1					
ASA‐PS	classes 2–3	37	1.14	0.692–1.891	0.600			
	classes 0–1	44	1					
Surgery after chemotherapy	yes	15	0.6	0.314–1.163	0.132			
	no	66	1					
ALT^a^	≥32 IU/L	40	1.27	0.761–2.124	0.359			
	<32 IU/L	40	1					
GGT^a^	≥226 IU/L	39	1.64	0.972–2.759	0.064			
	<226 IU/L	39	1					
T‐Bil^a^	≥1.0 mg/dL	41	1.39	0.832–2.301	0.209			
	<1.0 mg/dL	39	1					
Albumin^a^	≥3.4 g/dL	40	1.6	0.949–2.713	0.078			
	<3.4 g/dL	39	1					
Neutrophil‐to‐lymphocyte ratio^a^	≥2.83	40	2.08	1.243–3.482	0.005	2.13	1.242–3.639	0.006
	<2.83	40	1			1		
CEA^a^	≥3.0 ng/mL	40	1.47	0.869–2.492	0.151			
	<3.0 ng/mL	40	1					
CA19‐9^a^	≥250.0 U/mL	40	1.28	0.472–1.299	0.343			
	<250.0 U/mL	40	1					
Bismuth‐Corlette type	IIIa+IV	45	1.19	0.717–1.959	0.507	1.65	0.960–2.825	0.070
	I+II+IIIb	36	1			1		
Clinical metastasis^a^	yes	35	1.67	1.006–2.756	0.047	2.32	1.310–4.126	0.004
	no	45	1			1		
Clinical success^a^	yes	61	0.98	0.540–1.769	0.939			
	no	16	1					
Time to first reintervention	≥167 days	30	0.39	0.219–0.684	0.001	0.27	0.145–0.504	0.001
	<167 days	51	1			1		
EST (including previous)^a^	yes	28	0.67	0.379–1.173	0.667			
	no	52	1					
ENBD before chemotherapy induction	yes	54	0.95	0.558–1.604	0.836			
	no	27	1					
Cholangitis before chemotherapy induction, *n*	yes	48	1.28	0.764–2.152	0.346			
	no	33	1					
Drainage segments at chemotherapy induction, *n*	multiple	34	0.96	0.568–1.633	0.890			
	one segment	47	1					
Stenting above vs across the Sphincter of Oddi^a^	above	33	1.43	0.817–2.488	0.212			
	across	46	1					
SEMS vs. PS^a^	SEMS	7	1.19	0.464–3.030	0.722			
	PS	68	1					

ALT, alanine transaminase; ASA‐PS, American Society of Anesthesiologists physical status; CA19‐9, carbohydrate antigen 19‐9; CEA, carcinoembryonic antigen; CI, confidence interval; ENBD, endoscopic nasobiliary drainage; EST, endoscopic sphincterotomy; GGT, gamma‐glutamyltransferase; HR, hazard ratio; PS, plastic stent deployed transpapillary; SEMS, self‐expandable metallic stent; T‐Bil, total bilirubin.

^a^ Patients without data or stenting are excluded.

### 3.3 Stenting Above the SO Prolonged the TTFR From First‐line Chemotherapy Induction

**FIGURE 3:**
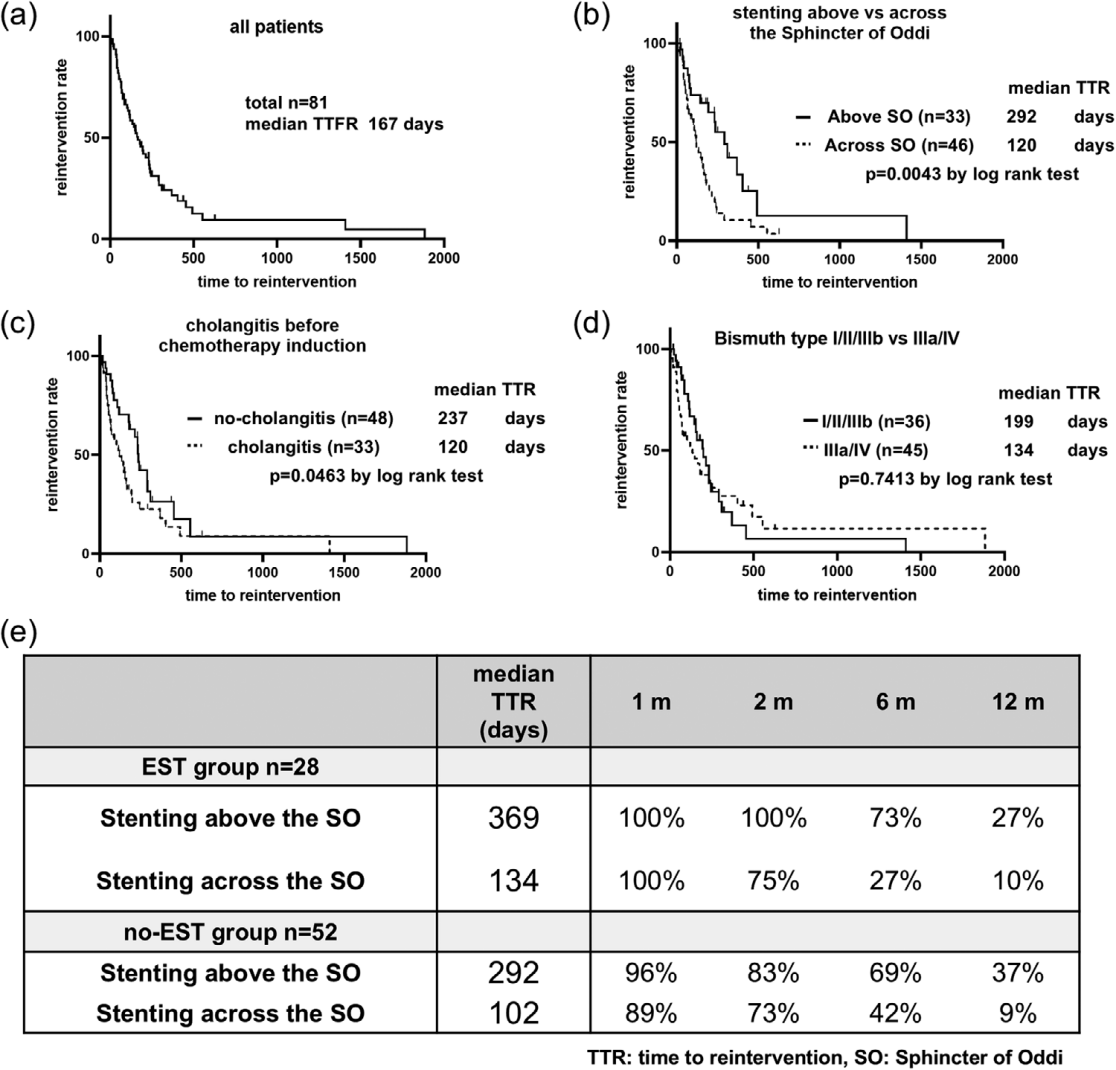
Factors related to the time to first reintervention (TTFR) from first‐line chemotherapy induction in the whole cohort and the no‐EST group. The TTFR of 81 patients with malignant hilar biliary obstruction (MHBO) was analyzed with the Kaplan–Meier method. (a) The median TTFR was 167 days. (b) Patients with stenting above the sphincter of Oddi (SO) had a longer TTFR than those with stenting across the SO (MST: 292 days vs. 120 days, *p* = 0.0043, log‐rank test). (c) Patients with cholangitis before chemotherapy induction had a shorter TTFR than those without cholangitis (MST: 120 days vs. 237 days, *p* = 0.0463, log‐rank test). (d) The TTFR did not differ between patients with Bismuth I/II/IIIB and those with Bismuth IIIa/IV disease (MST: 199 days vs. 134 days, *p* = 0.7413, log‐rank test). (e) The stent patency rate at 1–2 months was similar with stenting above and across the SO in both the EST and no‐EST groups. Compared with stenting across the SO, stenting above the SO achieved greater stent patency at 6–12 months after chemotherapy induction in both the EST group and the no‐EST group.

The median TTFR was 167 days (Figure 3a). Univariate analysis revealed that stenting above the SO prolonged the TTFR, whereas cholangitis before chemotherapy induction shortened the TTFR (Table 3). Combination chemotherapy did not affect TTFR. Multivariate analysis using these two factors and Bismuth type revealed that stenting above the SO was the only significant factor (HR: 0.44, *p* = 0.006, Table 3). Stenting above the SO prolonged the TTFR (292 vs. 120 days, *p* = 0.004, Figure 3b), whereas cholangitis before chemotherapy induction shortened the TTFR (120 days vs. 237 days, *p* = 0.046, Figure 3c). The Bismuth type did not affect the TTFR (Figure 3d).

**TABLE 3:** Risk factors associated with the time to first reintervention (TTFR) from first‐line chemotherapy induction.

Variable			Univariate analysis			Multivariate analysis		
		*n*	HR	95% CI	*p*‐Value	HR	95% CI	*p*‐Value
Sex	male	54	0.99	0.558–1.775	0.987			
	female	27	1					
Age	≥75 years	32	1.36	0.785–2.333	0.276			
	<75 years	49	1					
ASA‐PS	Classes 2–3	37	1.17	0.685–1.984	0.572			
	Classes 0–1	44	1					
Chemotherapy regimen	combination	67	1.07	0.501–2.275	0.865			
	monotherapy	14	1					
ALT^a^	≥32 IU/L	40	1.12	0.658–1.919	0.670			
	<32 IU/L	40	1					
GGT^a^	≥226 IU/L	39	1.2	0.697–2.061	0.513			
	<226 IU/L	39	1					
T‐Bil^a^	≥1.0 mg/dL	41	0.88	0.517–1.512	0.653			
	<1.0 mg/dL	39	1					
Albumin^a^	≥3.4 g/dL	40	1.07	0.602–1.900	0.820			
	<3.4 g/dL	39	1					
Neutrophil‐to‐lymphocyte ratio^a^	≥2.83	40	1.24	0.726–2.120	0.431			
	<2.83	40	1					
Bismuth‐Corlette type	IIIa+IV	45	1.09	0.639–1.874	0.742	1.04	0.600–1.820	0.878
	I+II+IIIb	36	1			1		
Clinical metastasis^a^	yes	35	0.63	0.357–1.103	0.105			
	no	45	1					
Clinical success^a^	yes	61	0.67	0.364–1.244	0.206			
	no	16	1					
EST (including previous)^a^	yes	28	1.10	0.621–1.934	0.753			
	no	52	1					
ENBD before chemotherapy induction	yes	54	0.91	0.519–1.607	0.753			
	no	27	1					
Cholangitis before chemotherapy induction, n	yes	48	1.73	1.002–3.003	0.049	1.43	0.831–2.468	0.196
	no	33	1			1		
Drainage segments at chemotherapy induction, n	multiple	34	0.75	0.423–1.340	0.335			
	one segment	47	1					
Stenting above vs across the Sphincter of Oddi^a^	above	33	0.44	0.243–0.783	0.006	0.44	0.241–0.789	0.006
	across	46	1			1		
SEMS vs PS^a^	SEMS	7	0.90	0.380–2.120	0.807			
	PS	68	1					

ASA‐PS, American Society of Anesthesiologists physical status; CI, confidence interval; HR, hazard ratio; ENBD, endoscopic nasobiliary drainage; EST, endoscopic sphincterotomy; PS, plastic stent deployed transpapillary; SEMS, self‐expandable metallic stent; T‐Bil, total bilirubin.

^a^ Patients without data or stenting are excluded.

### 3.4 The Incidence of Cholangitis Before Chemotherapy Was Greater in the EST Group Than the no‐EST Group and Affected the TTFR Only in the no‐EST Group

EST did not affect the MST or TTFR (Figure S2a,b). Compared with the no‐EST group, the EST group showed a greater incidence of cholangitis before chemotherapy induction (Table S1). No differences were observed between the two groups in terms of stenting, whether above or across the SO, or drainage segments.

Next, patients with or without a previous history of EST were analyzed independently. Among the 28 patients with a history of EST, no significant factors associated with the TTFR were identified (Table S2). Among the 52 patients without a history of EST, cholangitis before chemotherapy (HR 2.05, *p* = 0.041), stenting above the SO (HR 0.47, *p* = 0.038), and drainage of multiple segments at chemotherapy induction (HR 0.42, *p* = 0.035) were factors related to the TTFR on univariate analysis. None of these factors showed significance on multivariate analysis (Table 4).

**TABLE 4:** Risk factors associated with the time to first reintervention (TTFR) from first‐line chemotherapy induction in the no‐endoscopic sphincterotomy (no‐EST) group.

Variable			Univariate analysis			Multivariate analysis		
		*n*	HR	95% CI	*p*‐Value	HR	95% C.I.	*p*‐Value
Sex	male	37	0.75	0.347–1.620	0.464			
	female	15	1					
Age	≥75 years	19	0.99	0.500–1.966	0.981			
	<75 years	33	1					
ASA‐PS	Class 2–3	27	1.47	0.760–2.858	0.251			
	Class 0–1	25	1					
ALT^a^	≥32 IU/L	28	1.11	0.565–2.197	0.755			
	<32 IU/L	23	1					
gGTP^a^	≥226 IU/L	26	1.07	0.540–2.132	0.841			
	<226 IU/L	24	1					
T‐Bil^a^	≥1.0 mg/dL	27	1.22	0.618–2.399	0.570			
	<1.0 mg/dL	24	1					
Albumin^a^	≥3.4 g/dL	27	0.99	0.507–1.948	0.985			
	<3.4 g/dL	22	1					
Neutrophil‐to‐lymphocyte ratio^a^	≥2.83	27	1.1	0.560–2.146	0.788			
	<2.83	24	1					
Bismuth‐Corlette type	IIIa+IV	30	1.3	0.66–2.636	0.458			
	I+II+IIIb	22	1					
Clinical success^a^	yes	43	0.52	0.230–1.172	0.114			
	no	8	1					
Cholangitis before chemotherapy induction	yes	29	2.05	1.031–4078	0.041	1.71	0.818–3.558	0.155
	no	23	1			1		
ENBD before chemotherapy induction	yes	36	0.71	0.341–1.474	0.708			
	no	16	1					
Drainage segments at chemotherapy induction^a^	multiple	19	0.42	0.195–0.943	0.035	0.5	0.221–1.120	0.092
	1 segment	30	1			1		
Stenting above vs across the Sphincter of Oddi^a^	above	24	0.47	0.235–0.960	0.038	0.59	0.281–1.223	0.155
	across	27	1			1		
SEMS or PS^a^	SEMS	3	0.54	0.125–2.309	0.404			
	PS	45	1					

ASA‐PS, American Society of Anesthesiologists physical status; CI, confidence interval; HR, hazard ratio; ENBD, endoscopic nasobiliary drainage; EST, endoscopic sphincterotomy; PS, plastic stent deployed transpapillary; SEMS, self‐expandable metallic stent; T‐Bil, total bilirubin.

^a^ Patients without data or stenting are excluded.

Stenting above or across the SO, with or without EST, resulted in similar stent patency rates at 2 months, with patency rates of 89%–100% at 1 month and 73%–100% at 2 months. At 6 and 12 months, stent patency was greater in the above‐the‐SO group regardless of EST, with patency rates of 69%–73% (vs. 27–42%) at 6 months and 27%–37% (vs. 9%–10%) at 12 months (Figure 3e).

## 4 Discussion

In this study, cholangitis and stenting across the SO affected the TTFR. Univariate analysis revealed that cholangitis before chemotherapy induction affected the TTFR in the total cohort and in the no‐EST group, but this effect was not detected in the EST group (Tables 3 and 4; Table S2). Cholangitis was less frequent in the no‐EST group with a preserved SO with antireflux function (52%, 27/52) than in the EST group (75%, 21/28) (Table S1). Consequently, avoiding cholangitis may be a more critical factor in the no‐EST group than in the EST group. We have shown that deploying stents above the SO prolonged the TTFR only in the no‐EST group and not in the EST group. In a meta‐analysis of plastic stent deployment for malignant biliary obstruction, compared with stenting across the SO, stenting above the SO resulted in a longer overall patency time (HR 0.60, *p* = 0.001) [4]. However, our data revealed that neither cholangitis nor stenting above the SO affected the TTFR in the EST group. The lack of significance detected in the EST group may be attributed to the small sample size and, in part, to the nonfunctional SO in the EST group, where the risk of biliary infection is unavoidable, making stenting above the SO insufficient to prevent cholangitis and prolong the TTFR in the EST group.

The TTFR may reflect the tumor response to chemotherapy. The TTFR was correlated with OS in patients with MHBO (Figure 2e). This factor affected prognosis independent of clinical metastasis, the NLR, and the Bismuth type (Table 2). Sixty out of 81 patients in this study received cisplatin‐/gemcitabine‐based regimens. The median progression‐free survival of patients treated with the cisplatin‐gemcitabine regimen was reported to be 8.0 months in the ABC‐02 trial [13], which was longer than the median TTFR of 5.6 months in our study. However, the horizontal progression of MHBO is difficult to detect under stent deployment, even via multidetector CT, because of imaging artifacts. In our study, only 11% (6/56, Table 1) had unscheduled reintervention due to tumor progression. In addition, the TTFR was correlated with the first‐line treatment duration, which we consider a surrogate marker for progression‐free survival (Figure 2f). In the no‐EST group, where the effect of cholangitis was limited because of its low incidence, stenting of multiple segments was a factor prolonging the TTFR according to the univariate analysis (Table 4). When cholangitis is under control, and its effect on the TTFR is low, the TTFR may reflect horizontal tumor progression yet to be detectable by CT.

Stenting for patients receiving NAC may differ from that for patients receiving palliative therapy or upfront surgery. At our institution, we initially deploy unilateral plastic stents during first‐line chemotherapy to facilitate reintervention and prepare for possible conversion surgery. For patients intended to undergo surgery, unilateral drainage of the residual lobe is recommended to increase the future remnant liver volume [1, 2]. In our cohort, 88% (70/81) of the patients received plastic stents, and 58% (47/81) underwent drainage of one segment (Table 1). In pancreatic cancer, for which NAC is the standard therapy in Japan, metal stents have been shown to be superior to plastic stents in a meta‐analysis of inoperable pancreatic cancer patients, with a 4.45‐month longer stent patency, lower complication rates, and fewer reinterventions [18]. In contrast, a recent study in biliary cancer suggested that when deployed above the SO, plastic stents and metal stents have equal clinical success rates, median times to recurrent biliary obstruction, and adverse event rates [6]. We started chemotherapy with stenting of one segment in 58% of the patients. NAC for pancreatic cancer is performed for 2–6 months, depending on the local invasion of the primary tumor [19]. In our analysis, stenting above or across the SO, either with or without EST, resulted in similar stent patency rates (73%–100%) at 2 months (Figure 3e). However, at 6 and 12 months, the stent patency rate was greater after stenting above the SO (69%–73%) than across the SO (27%–42%), regardless of EST (Figure 3e). In 15 surgical cases (median NAC: 65 days), PS above the SO reduced stent trouble (33% vs. 78% for PS across SO). NAC‐to‐surgery intervals did not differ significantly by reintervention status (91 vs. 83 days, *p* = 0.79). In a meta‐analysis of biliary tract cancer, the time from diagnosis to surgery was 172 days in the NAC group and 25 days in the upfront surgery group [9]. Patients for whom NAC is planned to continue for more than 2 months may benefit from stenting above the SO, regardless of EST.

Our study has several limitations. First, this was a single‐center retrospective study. Chemotherapy regimens varied among patients. Patients with different cancer types, including gall bladder carcinoma, intrahepatic cholangiocarcinoma, and perihilar cholangiocarcinoma, were analyzed as a single group. For future analysis, a multicenter prospective study is warranted. Second, the extent of EST varied among patients, and it remains unclear which patients in the EST group had lost the function of the SO.

In conclusion, among patients with MHBO receiving systemic therapy, stenting above the SO was associated with a longer TTFR, particularly in those without prior EST and in those expected to continue chemotherapy for more than 2 months; these findings suggest that, in surgery‐intended and NAC candidacy settings, this approach may be considered a practical option.

## Author Contributions

**Katsuhiko Sato**, conceptualization (lead); investigation (lead); methodology (lead); formal analysis (lead); supervision (lead). **Minoru Shigekawa**, conceptualization (equal); methodology (equal); supervision (equal). **Teppei Yoshioka**, conceptualization (equal). **Kazuma Daiku**, methodology (equal). **Syuhei Yamamoto**, methodology (equal); formal analysis (equal). **Yu Sato**, conceptualization (equal); methodology (equal). **Takahiro Kodama**, conceptualization (equal); methodology (equal). **Hayato Hikita**, supervision (equal). formal analysis (equal). **Tetsuo Takehara**, conceptualization (equal); supervision (equal); formal Analysis (equal).

## Funding

The authors have nothing to report.

## Ethics Statement

This study was approved by the Institutional Review Board of The University of Osaka Hospital (approval number: 20565). This retrospective study employed an opt‐out approach, and therefore, the requirement for written informed consent was waived.

## Conflicts of Interest

The authors declare no conflicts of interest.

## Supporting information




**FIGURE S1**: Biliary drainage strategy for malignant hilar biliary obstruction in this study.

**FIGURE S2**: Effect of EST on overall survival (OS) and the time to first reintervention (TTFR) from first‐line chemotherapy induction. (a) The median OS of the whole cohort of 81 patients in the EST group and the no‐EST group was 580 days and 436 days, respectively (*p* = 0.1036, log‐rank test). (b) The median TTFR in the EST group and no‐EST group was 177 days and 154 days, respectively (*p* = 0.2550, log‐rank test).

**TABLE S1**: Comparison of the EST and no‐EST groups.

**TABLE S2**: Risk factors associated with the time to first reintervention (TTFR) from first‐line chemotherapy induction in the EST group.

**TABLE S3**: Effect of post ERCP pancreatis on chemotherapy induction.
