# Supplementary figures and images for: Optimal Stenting Strategy During Chemotherapy: Impact of Time to First Reintervention on Survival in Malignant Hilar Biliary Obstruction
Source: DEN Open. 2026 May 23;7(1):e70351. doi: 10.1002/deo2.70351 (PMC13240403; doi:10.1002/deo2.70351)

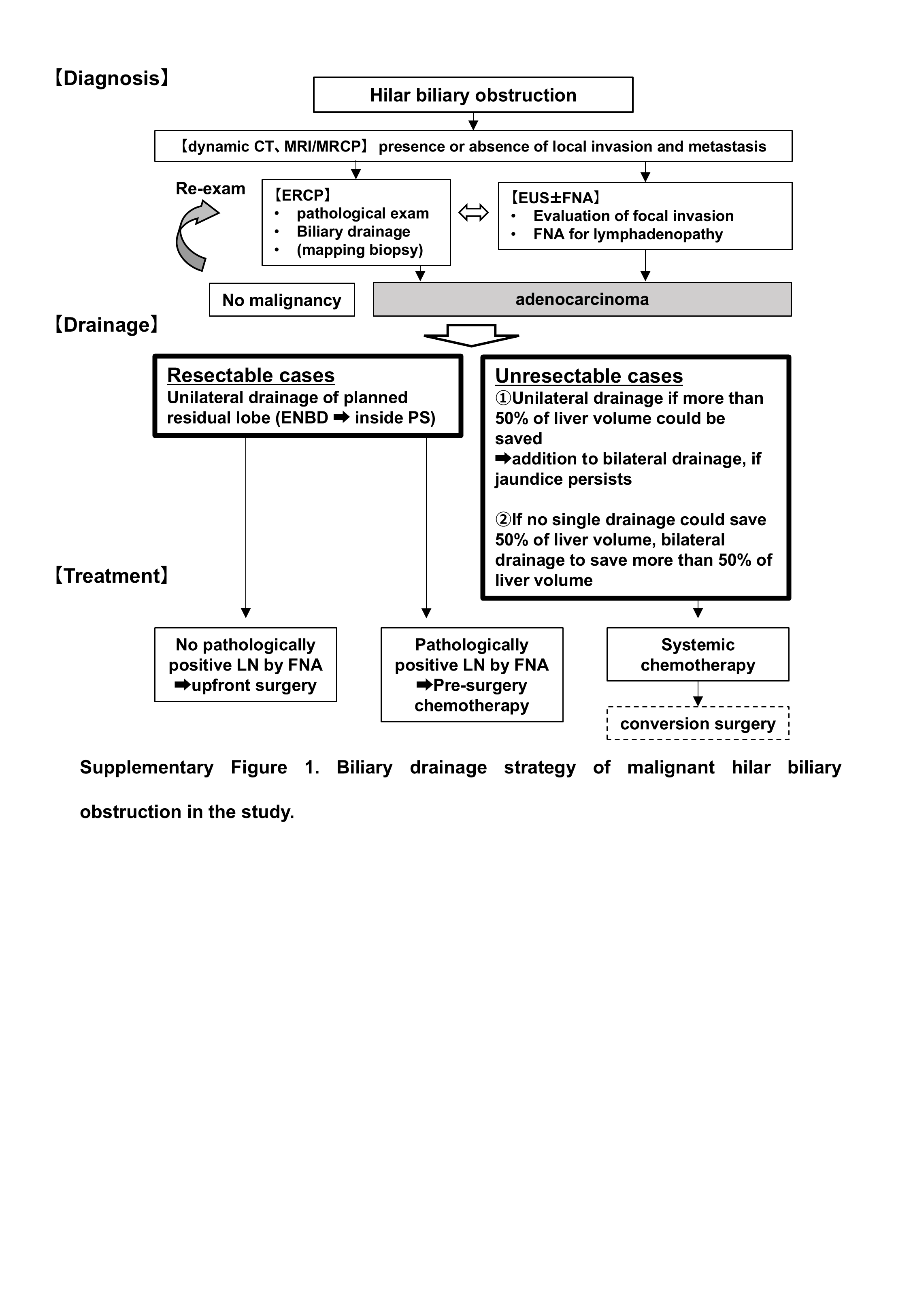

Supplement: Supplementary file 1 — FIGURE S1: Biliary drainage strategy for malignant hilar biliary obstruction in this study. [file DEO2-7-e70351-s005.TIF]

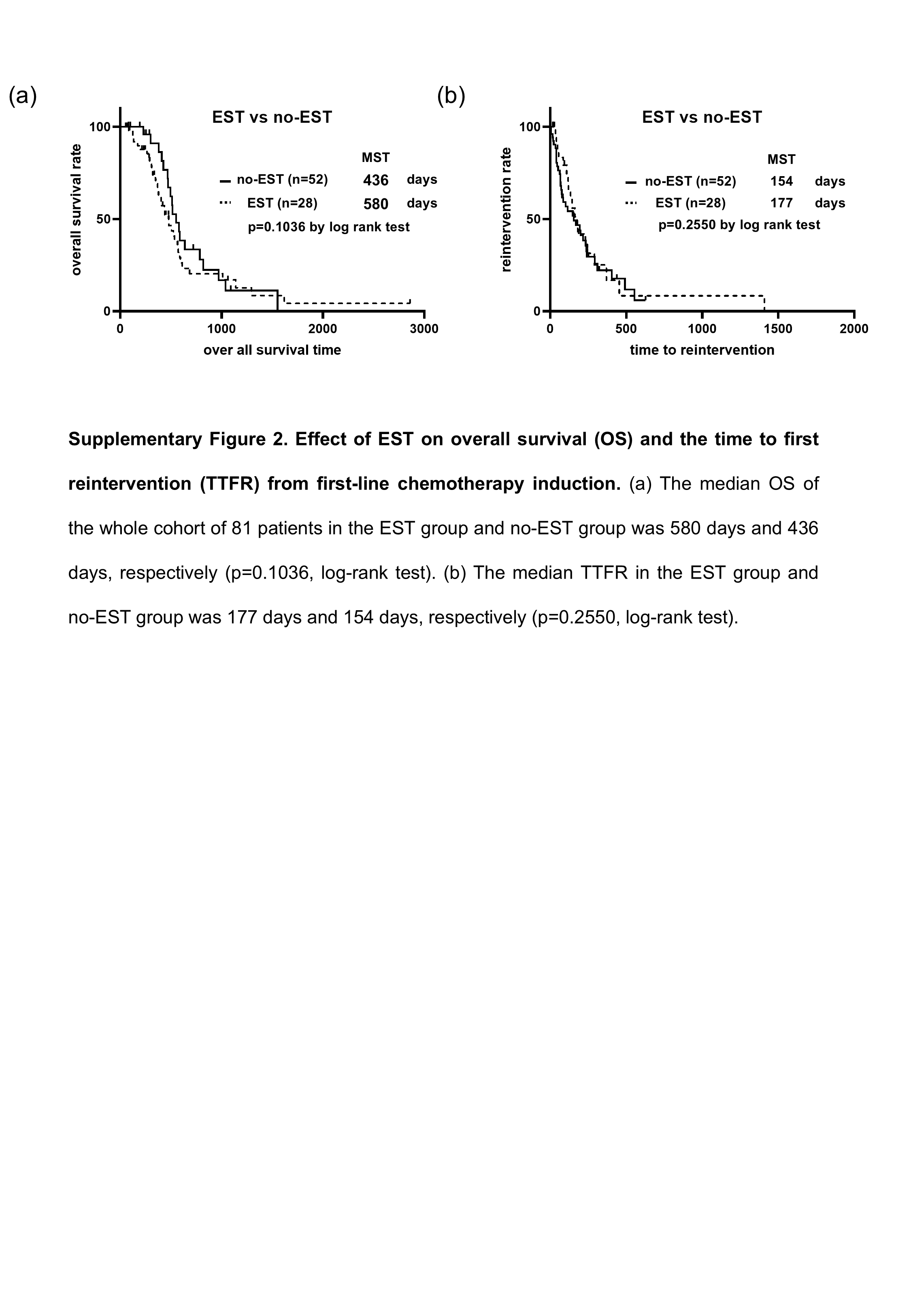

Supplement: Supplementary file 2 — FIGURE S2: Effect of EST on overall survival (OS) and the time to first reintervention (TTFR) from first‐line chemotherapy induction. (a) The median OS of the whole cohort of 81 patients in the EST group and the no‐EST group was 580 days and 436 days, respectively (p = 0.1036, log‐rank test). (b) The median TTFR in the EST group and no‐EST group was 177 days and 154 days, respectively (p = 0.2550, log‐rank test). [file DEO2-7-e70351-s003.TIF]
